# Effects of different virtual reality technology driven dual-tasking paradigms on posture and saccadic eye movements in healthy older adults

**DOI:** 10.1038/s41598-022-21346-6

**Published:** 2022-10-27

**Authors:** Yu Imaoka, Andri Flury, Laura Hauri, Eling D. de Bruin

**Affiliations:** 1 Motor Control and Learning Laboratory, Institute of Human Movement Sciences and Sport, Department of Health Sciences and Technology, ETH Zurich, 8093 Zurich, Switzerland; 2 Division of Physiotherapy, Department of Neurobiology, Care Sciences and Society, Karolinska Institute, 141 83 Stockholm, Sweden; 3 School of Health Professions, Eastern Switzerland University of Applied Sciences, 9001 St. Gallen, Switzerland

**Keywords:** Biomedical engineering, Biomechanics, Alzheimer's disease, Prognostic markers, Saccades

## Abstract

Postural sway and eye movements are potential biomarkers for dementia screening. Assessing the two movements comprehensively could improve the understanding of complicated syndrome for more accurate screening. The purpose of this research is to evaluate the effects of comprehensive assessment in healthy older adults (OA), using a novel concurrent comprehensive assessment system consisting of stabilometer and virtual reality headset. 20 healthy OA (70.4 ± 4.9 years) were recruited. Using a cross-sectional study design, this study investigated the effects of various dual-tasking paradigms with integrated tasks of visuospatial memory (VM), spatial orientation (SO), and visual challenge on posture and saccades. Dual-task paradigms with VM and SO affected the saccadic eye movements significantly. Two highly intensive tests of anti-saccade with VM task and pro-saccade with SO task also influenced postural sway significantly. Strong associations were seen between postural sway and eye movements for the conditions where the two movements theoretically shared common neural pathways in the brain, and vice versa. This study suggests that assessing posture and saccades with the integrated tasks comprehensively and simultaneously could be useful to explain different functions of the brain. The results warrant a cross-sectional study in OA with and without dementia to explore differences between these groups.

## Introduction

Dementia is a relevant neurodegenerative disorder in a growing ageing society. Since no effective cure is currently available and research has increasingly shown that dementia may be preventable, early diagnosis and intervention are important especially at the early stage of mild cognitive impairments (MCI)^1,2^. Due to the complicated syndrome of dementia, a comprehensive and multidisciplinary diagnostic approach is expected to assess the disorder more precisely^3–5^. For example, research analysed various neuroimaging data and clinical scores comprehensively, integrating a machine learning algorithm^6^. The study found a higher classification of MCI compared to a single assessment. Similarly, a study reported that a multimodal approach analysing behavioural and physiological data could improve the assessment of early dementia^7^. However, while a comprehensive assessment approach using multiple biomarkers and machine learning has the potential to achieve more precise detection of dementia, more computational capacity is required accordingly^8^. High computational cost leads to more expensive hardware configuration and more time-consuming signal processing. Considering a simple and time-efficient screening tool is in demand in daily clinical practice, more computationally efficient methods need to be considered.

Among various prospective biomarkers for dementia screening, movement behaviours could be potentially useful for the efficient comprehensive assessment. Specifically, previous studies have found that postural sway and saccadic eye movements were significantly affected in a MCI population. For example, increasing postural sway speed especially in anterior–posterior (AP) direction could be a discriminator of MCI^9,10^. The research suggested that people with MCI may have impairment in processing visual information, causing more postural sway. A study also evaluated the influence of dynamic visual stimuli from a projector on posture in healthy older adults (OA) and people with MCI. The study observed that people with MCI had a significant increase in postural sway during the post-stimulus period, implying that the MCI group impaired their adaptation function of posture to dynamic visual stimuli^11^. Likewise, saccadic dysfunction could be a differentiator of MCI. Prior research analysed latency and error rate of anti-saccade task in people diagnosed with amnestic MCI and non-amnestic MCI^12^. The results showed that the amnestic MCI group had longer latency and more error rate than the non-amnestic MCI group. It also found a strong association between the increasing saccade error rate and lower score of memory testing. While some overlapping brain areas are used for both locomotion and saccades, neural networks differ greatly between these two movements^13^. It is also reported that there could be a limitation of a single assessment due to multiple factors contributing to postural instability and impaired saccadic eye movements^14,15^. Therefore, similar to research that found unique interactions between facial and eye movements in people with Alzheimer’s disease (AD)^16^, investigating the two movements of posture and saccades comprehensively could indirectly evaluate wider regions of the brain and thus improve the understanding of brain activities for more accurate dementia screening.

To enhance the comprehensive assessment of posture and saccades further, head-mounted display (HMD) virtual reality (VR) technology is likely to be useful. Assuming that HMD VR technology could improve the sensitivity of postural sway assessment for dementia screening^17^, we developed a combined assessment system consisting of stabilometer and VR headset^18^. We discovered that healthy OA swayed more in anterior–posterior direction when they were intentionally exposed to a visual flow moving forwards and backwards in VR environments compared to eyes-open condition without the VR headset. As another study reported^19^, we also observed that the system was feasible for healthy OA. Therefore, the postural assessment integrating HMD-based VR technology could provoke different postural sway and be useful in detecting small distinct postural instabilities in people with dementia. Similarly, a VR headset with integrated eye-trackers could enhance an ocular assessment. We created a new assessment system of saccadic eye movements, using the VR headset with eye-trackers, VIVE Pro Eye (HTC Corporation, Taiwan)^20^. We designed new software (https://github.com/MotorControlLearning/SaccadeVR-mobile) to record eye movements while displaying VR environments for saccade assessment based on the previously proposed standardised saccade protocol^21^. Experiments with young adults revealed that the device can function as a saccade assessment tool. Thus HMD-based VR technology could upgrade both assessments of posture and saccades.

With the hypothesis that a comprehensive assessment of postural sway and saccadic eye movements powered by HMD VR technology could increase the screening accuracy of dementia, we have developed a novel concurrent comprehensive assessment system of postural and ocular movements in this study. We combined the stabilometer and HMD with eye-trackers based on our previous studies^18,20^. We have also integrated additional tasks of visuospatial memory (VM), spatial orientation (SO), and visual challenge (VC) into our HMD VR-based standard saccade assessment system^20^. We assumed that saccadic eye movements measured in these different VR environments could signify the functions of integrated tasks. A visuospatial memory test showed potential as a diagnostic tool because of the significantly poorer performance in individuals with AD^22,23^. Visuospatial memory deficits may be observed through eye movement analyses^24^. A spatial orientation test is another way to investigate people with dementia. In fact, an assessment of spatial navigation may be a better diagnosis tool than an episodic memory assessment since the deficits in spatial navigation possibly occur earlier than those in memory^25,26^. Specifically, assessment of the ability to switch from allocentric frame to egocentric frame could be a useful predictor. Since the hippocampus is more involved in the allocentric processing while the egocentric processing generally activates the medial parietal lobe, the switching task is likely to activate wider areas of the brain^27^. Furthermore, research reported the significant visual dysfunction under visual challenges in attention, contrast sensitivity, and stereopsis in people with AD, observing a strong association between the visual and cognitive functions^28–30^. Moreover, as the proposed new system can measure postural sway and eye movements simultaneously, the synchronised system would elucidate connections between postural sway and saccades and between diverse neural pathways of brain functioning more precisely^31,32^. Therefore, the newly designed concurrent comprehensive assessment of posture and saccades with the three integrated tasks could evaluate the diverse brain functions indirectly, elucidate the interplay between posture and saccades, and, thus, be potentially useful as a comprehensive assessment tool of dementia.

**Figure 1:**
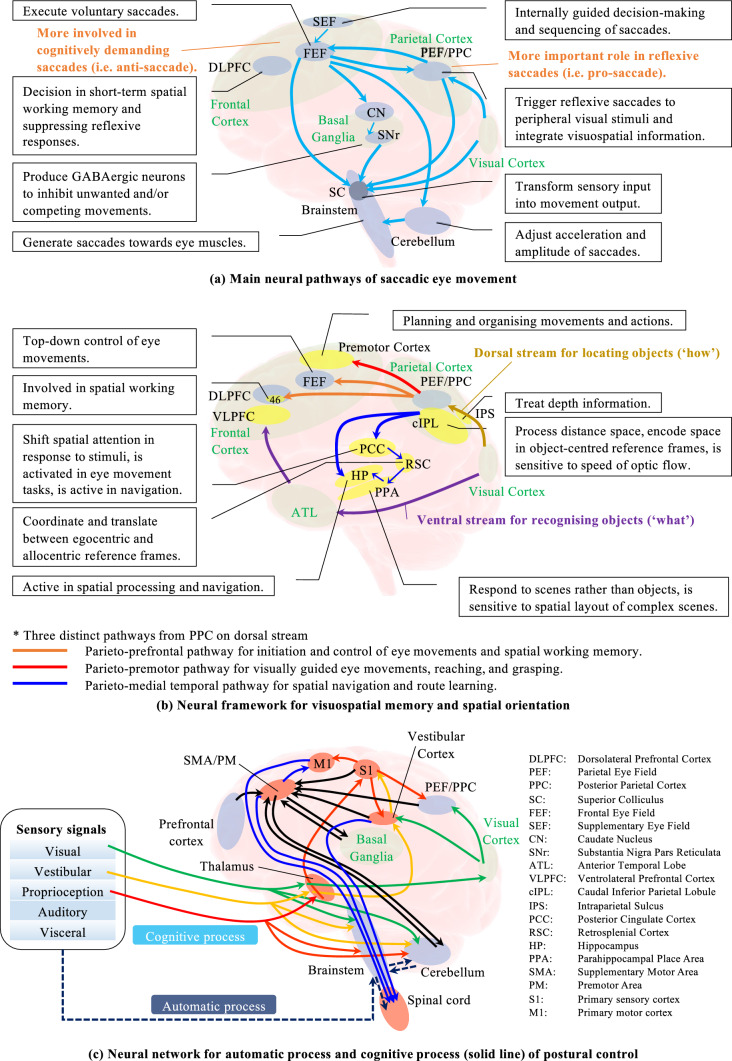
Neural pathways activated in three different activities. (**a**) Anti-saccade task activates the frontal lobe more, whereas pro-saccade task elicits rather the posterior parietal cortex. (**b**) The parieto-prefrontal pathway is used for visuospatial memory, while the parieto-medial temporal pathway is activated in spatial orientation. (**c**) The frontal lobe and parietal cortex are active in cognitive processing of postural control.

More specifically, Fig. 1 visualises the theoretical background of proposed integrative approach to examine the related neural pathways in the brain. Figure 1a illustrates the neural circuits used in pro-saccade and anti-saccade tasks. Two different cortices are mainly activated. The parietal eye field (PEF) is more active in making automatic or reflexive saccades (i.e., pro-saccade), while the frontal lobe (FL) plays a more important role in triggering cognitively demanding saccades (i.e., anti-saccade)^33,34^. The FL—basal ganglia (BG)—superior colliculus (SC) may also be crucial in conducting voluntary saccades. Figure 1b explains a neural framework of visuospatial processing. Two main streams are suggested: dorsal or “how” stream for locating objects and ventral or “what” stream for recognising objects^35,36^. Three major pathways diverge from the posterior parietal cortex (PPC) in the dorsal stream: (1) parieto-prefrontal pathway for spatial working memory, (2) parieto-premotor pathway for visually guided eye movement, and (3) parieto-medial temporal pathway for spatial navigation. Object working memory is related to the ventral pathway^36,37^. Figure 1c describes the basic neural network activated in postural control^38,39^. Automatic process of postural control mainly requires the brainstem and spinal cord to maintain an upright body posture against the gravity force, whereas the cognitive postural control involves the cerebral cortices to keep a posture in challenging environmental conditions.

In summary, HMD-VR based comprehensive assessment of posture and saccades with the integrated tasks of visuospatial memory, spatial orientation, and visual challenge could expand the scope of evaluation of the brain and, thus, improve the screening accuracy of dementia. To the best of our knowledge, this is the first study to develop the proposed concurrent comprehensive assessment system and to investigate the effects of various dual-tasking paradigms requiring posture and saccade controls with the integrated tasks. We primarily hypothesised that (1) saccadic eye movements would be affected by more demanding dual-task paradigms with the three integrated tasks and saccade performance could signify the functions of integrated tasks, (2) postural sway and saccadic eye movements could associate with each other in the conditions where common neural circuits are theoretically activated in the two movements, and (3) differences in magnitude of associations would be observed in saccade parameters between different test conditions since separate neural pathways were theoretically activated.

## Results

### Participants

After screening 23 participants with a health questionnaire and the Montreal Cognitive Assessment (MoCA), we evaluated 20 participants. Table 1 shows the demographic profile of the participants.

**Table 1:** Demographic profile of participants in the experiment.

Characteristic	Values
Gender	Men: 11, Women: 9
Age	70.4 ± 4.9 years
Weight	85.2 ± 22.6 kg
Height	172.0 ± 7.9 cm
BMI	28.4 ± 5.8 kg/m$^{2}$
Education	13.9 ± 2.9 years
MoCA score	27.2 ± 1.6
MoCA z-score (calculated by a formula derived from the normative data^40^)	0.15 ± 0.78

### Data characteristic

Levene’s test revealed $P\le 0.87$ and $P\le 0.81$ for the ocular and postural sway data respectively. Shapiro–Wilk test showed $P\le 0.001$ and $P<0.001$ for these data. Therefore, we used non-parametric statistics for the following analyses.

### Effects of dual-tasking paradigms on saccadic eye movements

**Figure 2:**
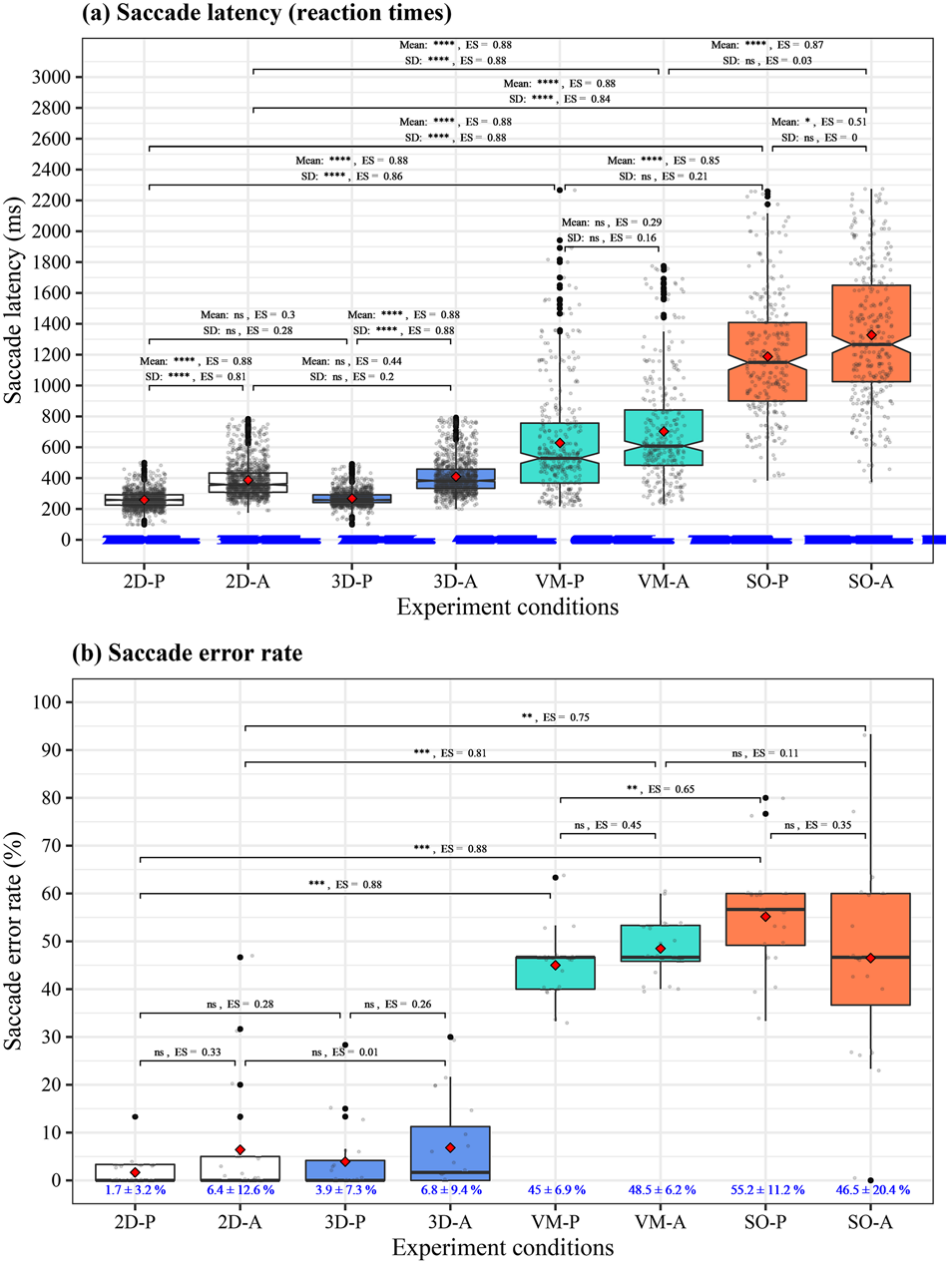
Distribution of saccade parameters of pro- and anti-saccades in 2D (baseline dual task condition), 3D (with visual challenge), visuospatial memory (VM), and spatial orientation (SO) VR environments. VM and SO tasks significantly affected (**a**) Latency (reaction times) and (**b**) Error rate. *P* Pro-saccade task, *A* Anti-saccade task.

We evaluated the effects of different dual-tasking paradigms on saccade parameters: mean and standard deviation (SD) of latency (reaction times), mean and SD of peak speed, and error rate for the conditions #4-11 (see Table 2). The results of non-parametric analysis of variance (ANOVA) show significant main effects of different VR environments on all the parameters except mean and SD of peak speed ($P<0.001$) and significant effects of different saccade tasks on mean and SD of each latency and peak speed ($P\le 0.002$). Post hoc analyses were conducted as shown in Fig. 2 for latency and error rate and in Supplementary Fig. S1 online for peak speed. *P* values and effect sizes (*ES*) were derived from Wilcoxon signed-rank test. We compared the data between different VR environments in the same pro- or anti-saccade task and between pro- and anti-saccade tasks in the same VR environment.

**Table 2:** Experimental conditions for concurrent comprehensive assessment of postural sway and saccadic eye movements with VR (*EO* eyes-open, *EC* eyes-closed, *VM* visuospatial memory, *SO* spatial orientation, *VC* visual challenge).

Test		Independent variables		Dual-tasking paradigms				
#	Name	VR environment	Oculomotor task	Posture	Saccade	VM	SO	VC
#1	EO	None	Gaze	$\circ$				
#2	EC	None	None	$\circ$				
#3	2D-G	2D	Gaze	$\circ$				
#4	2D-P (baseline dual-task)	2D	Pro-saccade	$\circ$	$\circ$			
#5	2D-A (baseline dual-task)	2D	Anti-saccade	$\circ$	$\circ$			
#6	3D-P	3D	Pro-saccade	$\circ$	$\circ$			$\circ$
#7	3D-A	3D	Anti-saccade	$\circ$	$\circ$			$\circ$
#8	VM-P	VM	Pro-saccade	$\circ$	$\circ$	$\circ$		
#9	VM-A	VM	Anti-saccade	$\circ$	$\circ$	$\circ$		
#10	SO-P	SO	Pro-saccade	$\circ$	$\circ$		$\circ$	
#11	SO-A	SO	Anti-saccade	$\circ$	$\circ$		$\circ$

Comparing the saccade latency between different dual-tasking paradigms in the same saccade task, we found that the effect of visual challenge was not significant in pro-saccade task between 2D and 3D VR environments (mean: $P=0.20$, $ES=0.30$; SD: $P=0.31$, $ES=0.28$) and in anti-saccade task (mean: $P=0.05$, $ES=0.44$, SD: $P=0.45$, $ES=0.20$). Significant effects of visuospatial memory and spatial orientation tasks were observed when compared to the baseline conditions with 2D VR environment (mean: $P<0.001$, $ES=0.88$; SD: $P<0.001$, $ES\ge 0.86$ in pro-saccade task and mean: $P<0.001$, $ES=0.88$; SD: $P<0.001$, $ES\ge 0.84$ in anti-saccade task). Next, when pro- and anti-saccade tasks were compared in each VR environment, we observed significant differences in 2D and 3D VR environments (mean: $P<0.001$, $ES=0.88$, SD: $P<0.001$, $ES\ge 0.81$). On the other hand, relatively small differences were seen in saccade tests with visuospatial memory task (mean: $P=0.20$, $ES=0.29$; SD: $P=0.56$, $ES=0.16$) and spatial orientation task (mean: $P=0.02$, $ES=0.51$; SD: $P\ge 0.99$, $ES=0$). We found significant differences in mean saccade latency between visuospatial memory condition and spatial orientation condition (mean: $P\le 0.001$, $ES\ge 0.85$), but not in variability of saccadic eye movement (SD: $P\ge 0.45$, $ES\le 0.21$).

Significant differences were not observed in saccade error rate between 2D and 3D VR environments in the same saccade task ($P\ge 0.14$, $ES\le 0.28$). The error rate increased in the conditions with visuospatial memory and spatial orientation tasks compared to the baseline conditions with 2D VR environment ($P\le 0.002$, $ES\ge 0.75$). Comparing the error rate between pro- and anti-saccade tasks in each VR environment, we did not find significant differences in all VR environments ($P\ge 0.09$, $ES\le 0.45$).

Unlike latency and error rate, peak speed was not significantly different between VR environments (mean: $P\ge 0.18$, $ES\le 0.38$; SD: $P\ge 0.17$, $ES\le 0.45$ in the pro-saccade task conditions and mean: $P\ge 0.14$, $ES\le 0.45$; SD: $P\ge 0.49$, $ES\le 0.27$ in the anti-saccade task conditions).

### Effects of dual-tasking paradigms on postural sway

**Figure 3:**
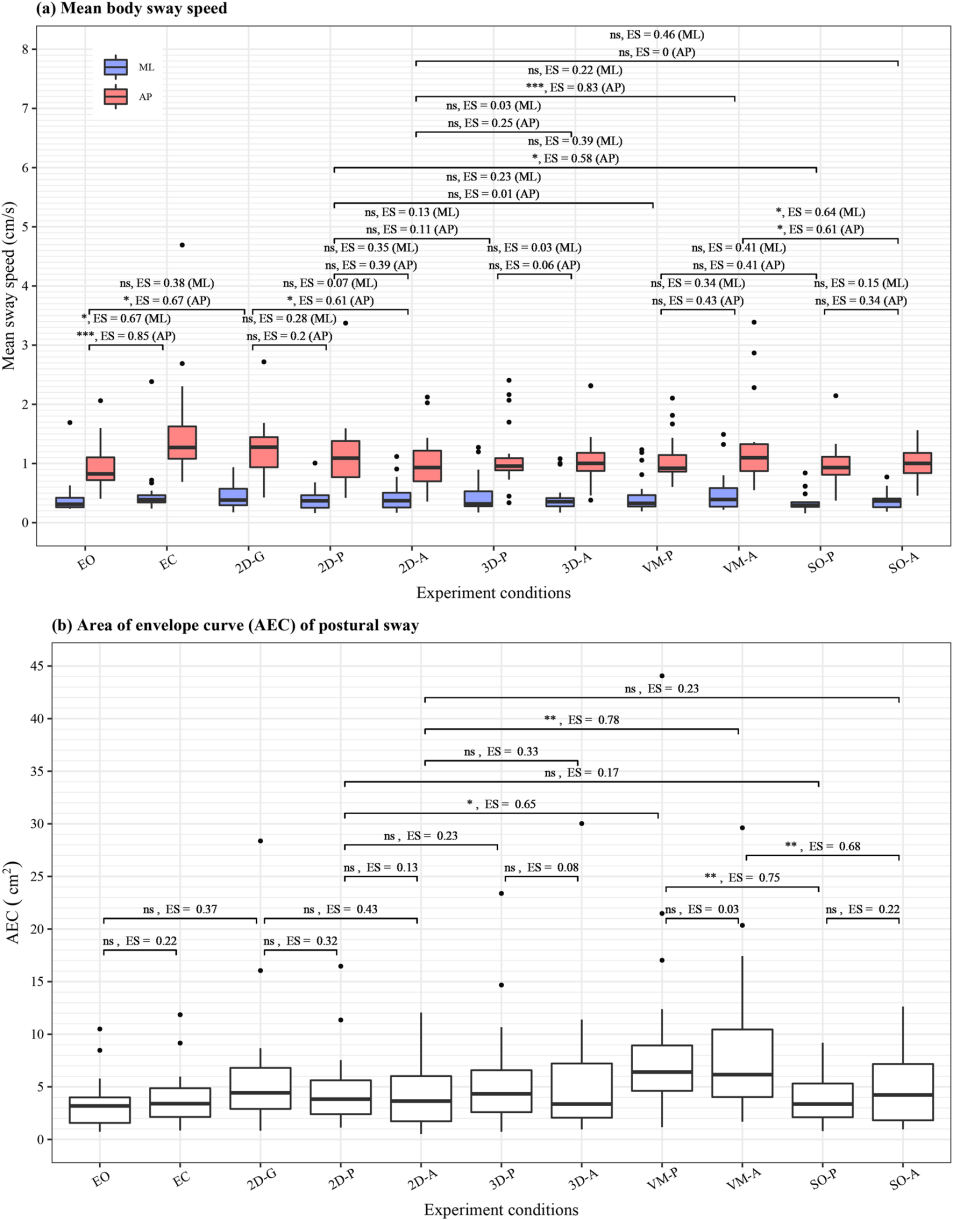
Distribution of postural sway in each test condition. (**a**) Mean postural sway speed, (**b**) AEC of postural sway in eyes-open, eyes-closed, 2D (baseline dual task condition), 3D (with visual challenge), visuospatial memory (VM), and spatial orientation (SO) VR environments. Significant differences were mainly seen in sway speed in anterior–posterior direction between (1) 2D-A and VM-A and (2) 2D-P and SO-P conditions. *EO* eyes-open, *EC* eyes-closed, *P* pro-saccade, *A* anti-saccade.

The ANOVA results revealed significant main effects of different VR environments on SD of centre of pressure (COP) displacement in anterior–posterior direction and sway area of envelope curve (AEC) ($P\le 0.002$). No significant effects of saccade types were observed ($P\ge 0.07$). Figure 3 visualises the data distribution of mean sway speed and AEC of all 11 test conditions. The figure also shows the results of post hoc analyses with *P* values and *ES* derived from Wilcoxon signed-rank test, comparing the baseline dual-task conditions and other conditions. Significant effects were seen in anterior–posterior mean sway speed for two cases: pro-saccade test with spatial orientation task ($P=0.04$, $ES=0.58$) and anti-saccade test with visuospatial memory task ($P<0.001$, $ES=0.83$). We also observed significant differences in AEC between the conditions with and without visuospatial memory task ($P=0.01$, $ES=0.65$ in the pro-saccade task conditions; $P=0.002$, $ES=0.78$ in the anti-saccade task conditions). Significant differences were not found for the case of visual challenge.

### Associations between saccades and posture

**Figure 4:**
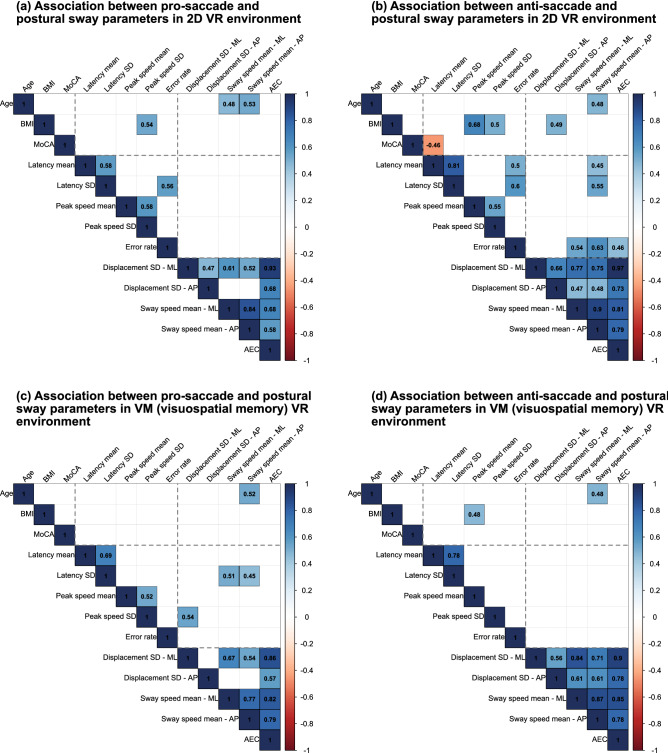
Correlation analysis between saccadic eye movements and postural sway in the baseline dual-task condition: (**a**) with pro-saccade task and (**b**) with anti-saccade task, and in dual-tasking paradigm with visuospatial memory (VM) task: (**c**) with pro-saccade task and (**d**) with anti-saccade task. More associations were observed in the baseline condition with anti-saccade task and the dual-task condition with visuospatial memory and pro-saccade tasks. More cognitively demanding tasks in these conditions could trigger the frontal lobe to be shared more commonly between saccade and posture controls.

We analysed the correlation between saccadic eye movements and postural sway in each of #4–11 conditions to investigate our hypothesis that related neural pathways would be shared commonly or used separately in maintaining posture and generating saccades depending on the different dual-tasking paradigms. Figure 4 visualises the association between demographic data, saccade parameters, and postural sway parameters in the conditions with and without visuospatial memory task (i.e. between 2D and VM VR environments). Associations in 3D and SO VR environments are also found in Supplementary Fig. S2 online. Only the associations at $5\%$ significance level were highlighted.

Almost no associations were found between posture and saccade parameters in the pro-saccade task conditions with 2D (baseline), 3D (visual challenge), and SO (spatial orientation) VR environments, whereas more associations were observed in the anti-saccade task conditions mainly between postural sway speed in anterior–posterior direction and saccade latency or error rate ($r_{s}\ge 0.45$).

Contrary to the findings in the other VR environments mentioned above, more associations were observed in the pro-saccade task condition with visuospatial memory task. On the other hand, no significant associations were seen in the anti-saccade task condition. Specifically, strong associations were seen between mean postural sway speed and SD of saccade latency ($r_{s}\ge 0.45$).

### Associations between test conditions

We explored the correlations of saccade parameters (mean and SD of latency, and error rate) and posture parameters (SD of displacement, mean sway speed, and sway area) between #4–11 conditions. Associations were highlighted at $5\%$ significance level in Supplementary Figs. S3 and S4 online for saccade and posture parameters respectively. More associations were found between the baseline condition and visual challenge condition ($r_{s}\ge 0.56$), whereas only a few associations were observed between the baseline condition and the conditions with visuospatial memory and spatial orientation tasks. No associations were seen in saccade performance between the conditions with visuospatial memory task and those with spatial orientation task. On the other hand, a large number of associations were found in most posture parameters between the conditions ($r_{s}\ge 0.45$).

## Discussion

We developed the novel concurrent comprehensive assessment system to measure posture and eye movements, using the stabilometer and HMD-based VR technology. With the hypothesis that the new system could indirectly assess the brain activities more broadly through the movement analyses, we evaluated the effects of different dual-tasking paradigms on saccadic eye movements and postural sway. We also investigated the associations between posture and saccade performances to understand the interplay between the two behaviours in each dual-task condition, and between the dual-tasking paradigms in each postural sway and saccadic eye movements.

First, the results of saccade assessment show that the dual-tasking paradigms with visuospatial memory and spatial orientation tasks caused the participants to take longer latency and more variability in initiating saccades. The test conditions also induced more saccade errors. However, the difference between pro- and anti-saccades was relatively small in the assessment paradigms. On the other hand, while no significant differences were observed between 2D and 3D VR environments in the same pro- or anti-saccade task, anti-saccade task caused significantly longer latency in each VR environment. The findings suggest that the additional tasks of visuospatial memory and spatial orientation probably require more cognitive resources than the visual challenge task, affecting the saccade performance more significantly compared to the performance in 2D and 3D VR environments. The visuospatial memory and spatial orientation tasks could be also more influential than the inhibitory control of anti-saccade on saccade performance because of small differences between pro-saccade and anti-saccade tasks in each VR environment. There was the clearer effect of anti-saccade task in 2D and 3D VR environments.

Second, contrary to the saccadic eye movements, the postural sway was rarely affected by the various dual-tasking paradigms. Nonetheless, test #9 with anti-saccade and visuospatial memory tasks affected the anterior–posterior sway speed and sway area significantly compared to the baseline dual-task conditions with 2D VR environment. The results indicate that the combination of anti-saccade and visuospatial memory tasks may trigger resource competition possibly because the inclusive tasks of visuospatial memory, anti-saccade, and cognitive postural control intensively activate neural pathways in the frontal lobe as illustrated in Fig. 1. The high intensity in the frontal lobe could hinder the posture maintenance in addition to the anti-saccade performance^41^. Likewise, test #10 with pro-saccade and spatial orientation tasks caused more postural sway in anterior–posterior direction compared to the baseline conditions with 2D VR environment. This may imply that the combination of pro-saccade and spatial orientation tasks induces more activities in the posterior parietal cortex (PPC), which is critical in spatial representation of objects for action planning and control^42^, and thus causes resource competition. The PPC is possibly shared between the tasks of pro-saccade, spatial orientation, and postural control (see Fig. 1). This competition would inhibit the postural control besides the pro-saccade performance. Significant effects were not observed on postural sway in the other conditions probably because the attentional resources are widely dispersed across the brain or the visual challenge requires less resource of attention. The outcomes suggest that the more cognitively demanding dual-tasking paradigms: anti-saccade test with visuospatial memory task (test #9) and pro-saccade test with spatial orientation task (test #10) trigger more postural sway possibly due to the limited resources especially in the frontal lobe and the PPC respectively. Yet, the healthy OA are still likely to prioritise their attentional resources to posture maintenance rather than saccade generation, considering the significant effects of visuospatial memory and spatial orientation tasks on the saccadic eye movements^43^. Nevertheless, the participants could have swayed more because they kept standing for longer duration in the tests #9 and #10 when compared to the baseline conditions #4 and #5 with 2D VR environment (see Fig. 6). The different duration of standing could have induced fatigue and, thus, more postural sway^44^.

Third, the correlation analysis explains the interaction between saccadic eye movements and postural sway. We consider that more cognitive postural control would be required in the designed dual-tasking paradigms^38^. Our results may suggest that separate neural pathways would be used in the baseline condition with pro-saccade task (test #4), where the PPC is more activated for pro-saccade control and the frontal lobe is used for postural maintenance. On the other hand, common pathways are probably shared between the saccade and posture controls in the baseline condition with anti-saccade task (test #5) because anti-saccade task is likely to activate the frontal lobe more than pro-saccade task (see Fig. 1a). Similar results were also found in the correlation analysis of conditions with visual challenge and spatial orientation tasks probably because of the same reasons. However, more associations were found in the condition with pro-saccade task when the visuospatial memory task was performed (test #8). As we found a significant effect of visuospatial memory task on postural sway in this condition, the cognitive processing of short-term memory task could trigger more activities in the frontal lobe even when the less cognitively demanding pro-saccade task is integrated. This could lead to the association between saccadic eye movements and postural sway, which is also supposed to activate the frontal lobe. On the other hand, the additional demanding task of anti-saccade (test #9) might provoke the cognitive capacity limitation, causing the non-significant associations between ocular and posture parameters^45^. In fact, we supplementarily measured pupillary response during the measurement, finding the lower normalised power spectrum density of pupillary response in the condition with anti-saccade and visuospatial memory tasks compared to the condition with pro-saccade and visuospatial memory tasks (0.045 mm$^{2}$/Hz for pro-saccade, 0.040 mm$^{2}$/Hz for anti-saccade). The result of less pupil dilation may also indicate the overloaded capacity limits^46^. Overall, saccade latency and anterior–posterior postural sway speed seem to be the main indicators to show the relationships between saccadic eye movements and postural sway. Our outcomes imply that related neural pathways could be commonly shared or independently used in generating saccades and maintaining body balance, depending on the designed dual-tasking paradigms^13^.

Finally, the comparison of each saccade and posture performance between the different VR environments reveals how the diverse dual-tasking paradigms associated with each other. The results show that healthy OA are likely to perform the similar saccadic eye movements in 2D and 3D VR environments. This implies that common neural pathways could be activated in both VR environments. Nevertheless, almost no associations were seen in saccade performance between the baseline conditions with 2D VR environment and those with the visuospatial memory and spatial orientation tasks. We did also not find associations between the conditions with visuospatial memory task and those with spatial orientation task. These results may suggest that separate neural pathways are probably activated in saccade control because of the different dual-tasking paradigms as expected (see Fig. 1). Considering the correlation analysis results and the lengthy reaction times of saccades in the conditions with visuospatial memory and spatial orientation tasks (test #8–11), saccade parameters measured in the conditions could signify the functions of visuospatial memory and spatial orientation respectively. Notably, strong associations were found between saccade latency and pupillary response only in the condition with pro-saccade and visuospatial memory tasks (test #8). As research found increasing pupillary dilation along with demanding working memory task^46^, our results may indicate that the saccade latency measured in this condition could indicate the visuospatial memory function. On the other hand, the result of strong associations in the posture parameters between most of the dual-tasking paradigms may support the finding that healthy OA would prioritise the attentional resources to maintain their posture over saccade generations as discussed above.

**Figure 5:**
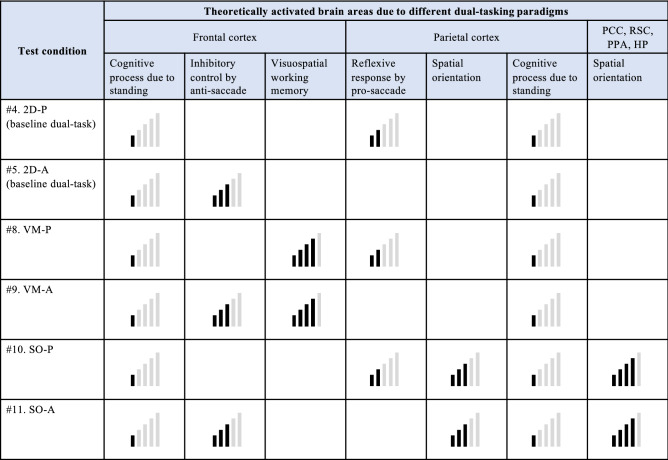
Dual-tasking assessment approaches formulated based on the findings of this study (symbols of signals illustrate which regions of the brain are activated theoretically in each test). Baseline assessment: conditions in 2D VR environment require basic dual-task of posture maintenance and saccadic eye movements: Test #4: Baseline condition with pro-saccade (2D-P) could be a simple comprehensive assessment to test the functions of frontal lobe and parietal cortex overall; Test #5: Baseline condition with anti-saccade (2D-A) could be a simple intensive test for the frontal lobe. Comprehensive assessment with an intensity in either the frontal lobe or the parietal cortex: Test #8: Pro-saccade test with visuospatial memory task (VM-P) would evaluate the frontal lobe and parietal cortex comprehensively, while requiring more intensity in the frontal lobe. Saccade latency and pupillary response could signify the function of visuospatial memory; Test #11: Anti-saccade test with spatial orientation task (SO-A) would also investigate the frontal lobe, parietal cortex, posterior cingulate cortex (PCC), retrosplenial cortex (RSC), parahippocampal place area (PPA), and hippocampus (HP) more comprehensively, while relatively requiring an intensity in the parietal cortex. Saccade latency could signify the function of spatial orientation. Highly intensive assessment in either the frontal lobe or the parietal cortex: Test #9: Anti-saccade test with visuospatial memory task (VM-A) could activate the frontal lobe more intensively, possibly reaching a maximum resource capacity to affect postural sway. Postural sway could indicate the possible overload in the frontal lobe; Test #10: Pro-saccade test with spatial orientation task (SO-P) would also intensify activities in the parietal cortex, possibly reaching a maximum resource capacity to affect postural sway. Postural sway could imply the maximum resource capacity in the parietal cortex.

To summarise, we have found that the newly developed concurrent comprehensive assessment system is effective to provoke different saccade and posture behaviours and to elucidate possible interplay between saccadic eye movements, posture, and integrated cognitive tasks of visuospatial memory and spatial orientation. Nevertheless, the additional visual challenge did not cause significant effects compared to the baseline conditions with 2D VR environment. Focusing on the baseline conditions and those with visuospatial memory and spatial orientation tasks, we have formulated dual-tasking assessment approaches based on the findings of this study. Figure 5 explains the brain areas theoretically activated due to the different dual-tasking paradigms and how each assessment method could be used in future studies. Overall, saccade latency, saccade error rate, postural sway speed, and postural sway area could be good indicators to show the effects of designed dual-tasking paradigms. The baseline condition with pro-saccade task (test #4) would be useful to test the functions of the frontal lobe and the parietal cortex comprehensively, while the baseline condition with anti-saccade (test #5) possibly checks the function of the frontal lobe more intensively. For more comprehensive testing with intensity on the frontal lobe, the integrated test of pro-saccade and visuospatial memory tasks (test #8) could be appropriate. Similarly, the combined test of anti-saccade and spatial orientation tasks (test #11) would meet the purpose of more comprehensive assessment with intensity on the parietal cortex. The saccade parameters could signify the integrated functions of visuospatial memory and spatial orientation. When inducing highly intense activities in either the frontal lobe or the parietal cortex, the dual-task paradigm with anti-saccade and visuospatial memory tasks (test #9) or the assessment with pro-saccade and spatial orientation tasks (test #10) would be suitable respectively. Postural sway behaviours are likely to indicate a maximum resource capacity, which is probably reached in these test conditions due to the high intensity in each cortex.

**Figure 6:**
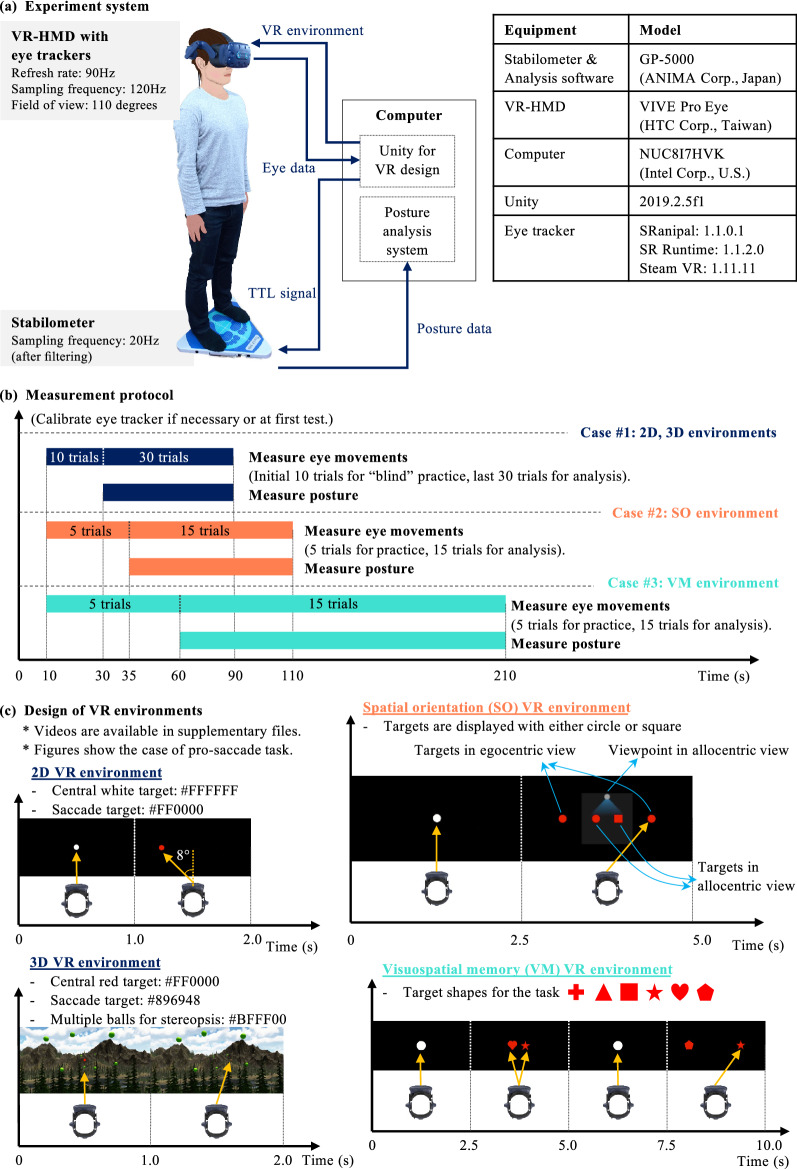
(**a**) Diagram of novel assessment system measuring postural sway and saccadic eye movements simultaneously; (**b**) Measurement protocol on time-domain; (**c**) Designs of four different VR environments.

Regardless of the findings, we acknowledge the limitations of our study and suggest some improvements. First, while we investigated the postural sway and saccadic eye movements, assuming that the non-invasive assessment could be useful to understand the neural pathways related to dementia symptoms (see Fig. 1), we did not measure them using more direct measures. For example, neuroimaging can inspect the activities of the frontal lobe and the parietal cortex more specifically. The specific neuropsychological assessments for visuospatial memory^47,48^ and spatial orientation functions^49^ are also useful to investigate brain functions clearly. With the direct assessments, we could investigate more diverse parameters to support the findings from this study^50,51^. Moreover, these direct measures could find potential neurological abnormalities of participants to refine our screening process. Second, we need to consider other possible factors affecting postural sway and saccadic eye movements. While the brainstem and the cerebellum play an important role in the motor control of both movements^13^, more studies have suggested the potential contribution of the cerebellum to cognitive processing^52,53^. In addition to more specific investigation of the brain areas such as the frontal lobe and the parietal lobe, future research needs to explore the cerebellar activities. The detailed analysis of the cerebellum would clarify the extent of its contribution to postural sway and saccadic eye movements and potentially show that subtle variations of these movements may be linked with cognitive impairment^54^. Third, we recognise that other elements could have affected the saccade performance. For example, circadian rhythms may have affected the saccadic eye movements, which require attention^55^. The saccade performance would be changed if we performed the assessment at the same time slot. Similarly, specific ophthalmological examination could have revealed potential dysfunction that could influence the saccadic eye movements^56^. Nevertheless, we prepared the baseline test conditions in this study to evaluate the effect of various dual-tasking paradigms rigorously. The comparison between the baseline conditions and the others would mitigate the impact of these possible systematic errors. Fourth, the experimental protocol can be improved. Future studies should implement a consistent number of saccade trials and measurement time in all conditions (Fig. 6b). The VR designs can be also updated. While each saccade trial was performed at a constant interval, expecting the relation between postural sway and saccadic eye movements on frequency domain^57^, the constant interval could have caused learning effects. The future system needs to consider the advantages of frequency response analysis. Finally, we need to increase the sample size and widen the scope of participants. The resulting MoCA score indicates that the participants were in a rather cognitively high functioning group of OA. Our research outcomes warrant future studies involving more OA with and without cognitive impairment in a wider range of cognitive functioning.

## Research method

### Study design

This study was organised at a local university. ETH Zurich ethics commission approved the ethics (2019-N-181). The study was carried out in accordance with ethical principles enunciated in the current version of the Declaration of Helsinki, the guidelines of Good Clinical Practice issued by ICH, and Swiss regulatory authority’s requirements. We recruited healthy OA aged 60 years and above. First, we explained the experimental protocol to the participants. If the participants agreed to join the measurement, we asked them to sign an informed consent and answer a health questionnaire (see Supplementary Fig. S5 online) to perform an initial screening on physical impairment and motion sickness. If the participants were successful in this first screening and neither physical impairment nor motion sickness was observed, their cognitive function was checked using the MoCA and compared to normative values^40^. Participants were included if their performance on the MoCA did not indicate cognitive impairment.

### Experimental protocol

Figure 6a shows the experimental set-up, mainly consisting of the stabilometer GP-5000 (ANIMA Corporation, Japan) and VR-HMD VIVE Pro Eye (HTC Corporation, Taiwan). Both devices were synchronised on Unity game engine. We hypothesised that dual-tasking paradigms with integrated tasks of visuospatial memory (VM), spatial orientation (SO), and visual challenge (VC) would provoke different eye movements. 11 test conditions were designed with four different VR environments as shown in Table 2: (#1) eyes-open (EO) without VR, (#2) eyes-closed (EC) without VR, (#3) gaze task in 2D VR environment (2D-G), (#4–5) pro-saccade or anti-saccade task in 2D VR environment, (#6–7) pro-saccade or anti-saccade task with visual challenge in 3D VR environment, (#8–9) pro-saccade or anti-saccade task with visuospatial memory task in VM VR environment, and (#10–11) pro-saccade or anti-saccade task with spatial orientation task in SO VR environment. Table 2 shows the list of experimental conditions and dual-tasking paradigms that we formulated. We defined the dual-task of saccadic eye movements and postural sway maintenance in 2D VR environments as baseline dual-task conditions. The other conditions with different VR environments have the additional loads of visual challenge, visuospatial memory, and spatial orientation. First, we measured only postural sway in the eyes-open and eyes-closed conditions for 60 s respectively. In the eyes-open condition, the participants looked at a cross mark at eye height and 1.5 m ahead. Next, we measured both posture and saccadic eye movements in the remaining conditions, the order of which was randomised. The participants were wearing the VR headset and glasses or contact lenses if they needed them to see the VR environments clearly. While wearing the VR headset, the participants stood on the stabilometer with feet placed at a comfortable width to eliminate the possible influence of weak lower-limb muscle strength^18^. We marked the foot position on the stabilometer with tapes to maintain the same foot position for each condition. The participants were also asked to keep their head stable to avoid possible effect of head movements on postural sway. The participants took a break if necessary between the trials.

Figure 6b explains the measurement protocol of concurrent measurement on time domain. First, we calibrated the eye-trackers if necessary and then started to record the eye movements. After the participants conducted a specific number of saccade trials: 10 for 2D and 3D environments, and 5 for visuospatial memory and spatial orientation environments, we started to measure postural sway. We prepared these trials for the participants to blindly practise saccadic eye movements and to eliminate the technical sampling issues^20^. The participants were not aware of the practice saccade trials and of when the posture recording started. Seamlessly, the participants continued to perform the rest of saccade trials: 30 for 2D and 3D environments, and 15 for visuospatial memory and spatial orientation environments, while their body balance was also measured.

Figure 6c illustrates the example of pro-saccade task for one cycle in each VR environment. The 2D VR environment consisted of black background and white and red targets^20^. The participants gazed at a white central target (#FFFFFF) for 1 s and then had to move their eyes towards the left or right within the next 1 s once the red target (#FF0000) appeared at 8$^{\circ }$ from the centre. In the gaze task, they kept gazing at the central white target. They followed the same protocol of saccade assessment in the 3D VR environment. We intentionally created the environment with visual challenge of more distractors with green spheres (#BFFF00), lower contrast of saccade targets (#896948), and stronger depth perception because these factors are possibly useful to evaluate cognitive functions of people with AD^28–30,58,59^. In the spatial orientation environment, the participants looked at a white central target (#FFFFFF) for 2.5 s and then saw egocentric and allocentric views in the next frame for another 2.5 s. We asked them to conduct a switching task from the allocentric frame to the egocentric frame and then perform saccades within the next 2.5 s. In this example, they looked at a small map from the allocentric view that displayed two red circle and square targets (#FF0000), understood the position of the red circle (2$^{\circ }$ from the centre) from the allocentric viewpoint, and moved their eyes towards the right red circle in the outer map from the egocentric view. In the visuospatial memory environment, one cycle consists of four frames. The participants gazed at a white central target (#FFFFFF) for 2.5 s, memorised two different red symbols (#FF0000) displayed at ± 2$^{\circ }$ from the centre within the next 2.5 s, looked at the central white target again for 2.5 s, and finally moved their eyes towards the right symbol that appeared in the second frame in this example. We used six symbols: cross, triangle, square, star, heart, and pentagon for the targets, referring to Test of Attentional Performance. The participants followed the same procedures in anti-saccade task but needed to move their eyes towards the opposite direction to the direction in pro-saccade task. We displayed the saccade targets on the left or right for equal number of times at ± 8$^{\circ }$ from the centre in all saccade tests. Potential saccadic intrusions were eliminated in the data processing by defining the eye movements that did not reach the amplitude of ± 1$^{\circ }$ as non-saccadic eye movements^20,60^. Inertial measurement unit (IMU) sensors embedded inside the VR headset were disabled to fix the view of VR scenery regardless of head movements. This setting mitigates the impact of possible head movements on saccade assessment. The video files of each VR environment are available in Supplementary Videos S1–S4 online.

### Signal processing

We processed the raw data of posture and eye movements on MATLAB R2019b (MathWorks, U.S.). Specifically, we computed the oculo-metrics: latency, peak speed, and error rate of each saccade trial, following the saccade detection algorithm^20^. We also calculated the duration of gaze at left and right targets in visuospatial memory and spatial orientation environments to improve the saccade detection algorithm. We assumed that the longest duration indicated the target that the participants selected. We then evaluated the mean and standard deviation (SD) of each saccade parameter. For body balance data, we calculated SD of centre of pressure (COP) displacement, mean body sway speed, and sway area of envelope curve (AEC) for all 11 test conditions.

### Statistical analysis

We analysed the calculated data statistically on R programming with version 4.0.2^61^. First, we performed Levene’s test and Shapiro–Wilk test to check the homogeneity of variance and the normality respectively for ocular and postural sway data. Second, following these tests, we performed non-parametric ANOVA to the test conditions of #4–11 (see Table 2) to evaluate the effects of different dual-tasking paradigms requiring postural control and ocular control assessed by saccades ($5\%$ significance level)^62^. Subsequently, we conducted Wilcoxon signed-rank test to investigate the *P* values and effect size (*ES*), by comparing each measurement parameter between the test conditions ($ES\ge 0.5$: large, $0.5\ge$ *ES* $\ge 0.3$: moderate, $0.3\ge$ *ES* $\ge 0.1$: small). Eyes-open, eyes-closed, and 2D-G conditions were also included for the post hoc analysis of postural sway. However, since eyes-open and eyes-closed conditions were without the VR headset, the weight of headset could be a factor influencing postural sway in the other conditions with the VR headset. Thus, we mainly evaluated the differences between the baseline dual-task conditions with 2D VR environment (tests #4 and #5) and the other test conditions with different dual-tasking paradigms of visual challenge, visuospatial memory, and spatial orientation (tests #6–11) to focus on the same hardware configuration. We adjusted the calculated *P* values due to multiple post hoc pairwise comparisons^63^. Third, we performed non-parametric Spearman rank correlation analysis to evaluate the association between ocular and postural sway parameters in each of #4–11 test conditions. Finally, to investigate the association between the test conditions #4–11 in each ocular and postural performance, we conducted non-parametric Spearman rank correlation analysis for each measurement parameter.

## Supplementary Information

Supplementary Figure S1.

Supplementary Figure S2.

Supplementary Figure S3.

Supplementary Figure S4.

Supplementary Figure S5.

Supplementary Video S1.

Supplementary Video S2.

Supplementary Video S3.

Supplementary Video S4.

## Data Availability

The datasets and specifications of VR designs generated during and/or analysed during the current study are available from the corresponding author on reasonable request.
