# Supplementary material for: Effects of different virtual reality technology driven dual-tasking paradigms on posture and saccadic eye movements in healthy older adults
Source: Sci Rep. 2022 Oct 27;12:18059. doi: 10.1038/s41598-022-21346-6 (PMC9613688; doi:10.1038/s41598-022-21346-6)
Supplement: Supplementary file 5 — Supplementary Figure S5. [file 41598_2022_21346_MOESM5_ESM.pdf]

Health Questionnaire

ETH
Eidgenössische Technische Hochschule Zürich
Swiss Federal Institute of Technology Zurich

**Part. ID:**	**Date:**	**Tester:**

1. General Questions

Gender:

☐ male ☐ female ☐ other:__________________________

Age: ____

Weight: ____

Height: ____

Years of education*: ____

* Primary and secondary school including apprenticeship or university

Do you smoke?

☐ No ☐ Yes, I smoke __________ (amount of) cigarettes per day.

Do you drink coffee?

☐ No ☐ Yes, 1-3 cups per day ☐ Yes, more than 3 cups per day

Do you drink alcohol?

☐ No ☐ Yes, 1-7 units per week ** ☐ Yes, more than 7 units per week **

** One unit is considered as one glass of wine (=2dl), one beer, or a small amount of high-proof alcohol

2. Questions about your health state

How would you rate your current health state?

☐ very good ☐ good ☐ fair ☐ poor ☐ I don't know

How would you rate your health state compared to peers of the same age?

☐ better ☐ same ☐ worse ☐ I don't know

How would you rate your balance?

☐ very good ☐ good ☐ fair ☐ poor ☐ I don't know

How would you rate your general muscle strength?

☐ very good ☐ good ☐ fair ☐ poor ☐ I don't know

Did your doctor diagnose you with one of the below mentioned diseases?

Diabetes mellitus	☐ yes	☐ no	☐ I don’t know
Polyneuropathy (nerve damage)	☐ yes	☐ no	☐ I don’t know
Hypertension (high blood pressure)	☐ yes	☐ no	☐ I don’t know
Heart failure	☐ yes	☐ no	☐ I don’t know
Heart attack	☐ yes	☐ no	☐ I don’t know
Stroke	☐ yes	☐ no	☐ I don’t know
Malignant tumor / cancer	☐ yes	☐ no	☐ I don’t know
Respiratory disorders (bronchial tubes)	☐ yes	☐ no	☐ I don’t know
Stomach or intestinal disorders	☐ yes	☐ no	☐ I don’t know
Joint diseases (rheumatism, arthrosis, gout)	☐ yes	☐ no	☐ I don’t know
Osteoporosis (bone loss)	☐ yes	☐ no	☐ I don’t know
Eye diseases	☐ yes	☐ no	☐ I don’t know

If yes, which one? ____

Which medication do you regularly take at the moment?

(type, frequency, dose; e.g. ASS 100, daily, 1 pill?

____
____
____
____
____

Do you have any pain?

☐ no pain ☐ less than daily ☐ daily

How stron is the pain (pain intensity)?

☐ little pain ☐ moderate pain ☐ sometimes very strong pain

Where do you have pain (localization)?

☐ back pain
☐ bone pain
☐chest pain during usual activity
☐ headache
☐ pain in the hip
☐ wound pain
☐ joint pain (except pain in the hip)
☐ muscle soreness
☐ stomach ache
☐ other localization: ____

Do you suffer from:

Hearing problems:	☐ yes	☐ no
Visual problems:	☐ yes	☐ no
Dizziness:	☐ yes	☐ no
Gait problems:	☐ yes	☐ no

3. Questions about mobility and physical activity

Do you have problems with your legs (wounds on the feet, varicose veins, arthrosis knee or hip pain), which limit the mobility?

☐ yes ☐ yes, sometimes ☐ no ☐ I don’t know

Do you use walking aids?

☐ none ☐ cane / stick / crutch ☐ rollator

Are you afraid of falling?

☐ no, never ☐ sometimes ☐ often ☐ yes, always

How many times did you fall in the last 6 months?

Number: ☐ 0 ☐ 1 ☐ >1
Why? (if known): ____

How often during the week do you do the following leisure / sport activities and for how long?

To go for a walk: ____
Fitness / exercise: ____
Gardening: ____
Others: ____

Do / did you play sports actively?

☐ yes ☐ no
Why did you stop? ____

Do / did you experience motion sickness on cars, trains, ships, airplanes, etc.?

☐ yes ☐ no
If yes, what vehicle(s) do / did you experience? ____

What is your main profession?

☐ sedentary work ☐ light physical work ☐ moderate physical work ☐ hard physical work

Thank you for answering the health questionnaire!
